# Efficacy of paliperidone palmitate 3-month formulation in preventing hospital admissions. 60 months of follow-up

**DOI:** 10.1192/j.eurpsy.2022.730

**Published:** 2022-09-01

**Authors:** S.L. Romero Guillena, B.O. Plasencia Garcia De Diego, F. Gotor Sanchez-Luengo

**Affiliations:** 1 UGC Salud Mental Virgen Macarena,, Psychiatry, seville, Spain; 2 Virgen del Rocio Hospital, Psychiatry, Seville, Spain

**Keywords:** hospital admissions, Paliperidone Palmitate 3-month formulation, schizophrénia, Efficacy

## Abstract

**Introduction:**

Paliperidone Palmitate 3-month formulation (PP3M) has shown a significantly longer time to relapse compared to placebo, with similar efficacy and safety to Paliperidone Palmitate 1-month (PP1M). However, studies of longer duration are required.

**Objectives:**

The main objective of this study is to determine the effectiveness of PP3M in the prevention of hospitalizations in patients with non-acute schizophrenia in a naturalistic outpatient psychiatric setting.

**Methods:**

Sample: 30 patients diagnosed with schizophrenia (DSM 5) that started treatment with PP3M after being stabilized with PP1M (the treatment dose was not changed in the four months before study inclusion) The mean dose of PP3M was 401. 55 mg Quarterly basis, the following evaluations were performed during a follow-up period of 60 months: The Clinical Global Impression-Schizophrenia scale (CGI-SCH) Treatment adherence, concomitant medication, and the number of hospitalizations. Efficacy values: Percentage of patients who remained free of admissions at the end of 60 months of follow-up. Other evaluation criteria: Average change from baseline visit to the final evaluation as assessed by score obtained on the following scale: GSI-SCH; percentage of patients on antipsychotic monotherapy, and treatment adherence rate.

**Results:**

The percentage of patients who remained free of admissions at the end of the 60 months was 83.25%. Mean variations from baseline scores at 60 months were: (-0.36 ±0-37) on the GCI-SCH. The rate of adherence to treatment with PP3M after 60 months was 86.58%.

**Conclusions:**

In our study, we found that paliperidone palmitate 3-month formulation effectively prevents admissions under daily clinical practice conditions.

**Disclosure:**

No significant relationships.

